# Knowledge, Attitude and Practice Regarding Dengue Fever among the Healthy Population of Highland and Lowland Communities in Central Nepal

**DOI:** 10.1371/journal.pone.0102028

**Published:** 2014-07-09

**Authors:** Meghnath Dhimal, Krishna Kumar Aryal, Mandira Lamichhane Dhimal, Ishan Gautam, Shanker Pratap Singh, Chop Lal Bhusal, Ulrich Kuch

**Affiliations:** 1 Nepal Health Research Council (NHRC), Ramshah Path, Kathmandu, Nepal; 2 Biodiversity and Climate Research Centre (BiK-F), Senckenberg Gesellschaft für Naturforschung, Frankfurt am Main, Germany; 3 Institute for Atmosphere and Environment, Goethe University, Frankfurt am Main, Germany; 4 Faculty of Social Sciences, Goethe University, Frankfurt am Main, Germany; 5 Natural History Museum, Tribhuvan University, Swoyambhu, Kathmandu, Nepal; CDC, United States of America

## Abstract

**Background:**

Dengue fever (DF) is the most rapidly spreading mosquito-borne viral disease in the world. In this decade it has expanded to new countries and from urban to rural areas. Nepal was regarded DF free until 2004. Since then dengue virus (DENV) has rapidly expanded its range even in mountain regions of Nepal, and major outbreaks occurred in 2006 and 2010. However, no data on the local knowledge, attitude and practice (KAP) of DF in Nepal exist although such information is required for prevention and control measures.

**Methods:**

We conducted a community based cross-sectional survey in five districts of central Nepal between September 2011 and February 2012. We collected information on the socio-demographic characteristics of the participants and their knowledge, attitude and practice regarding DF using a structured questionnaire. We then statistically compared highland and lowland communities to identify possible causes of observed differences.

**Principal Findings:**

Out of 589 individuals interviewed, 77% had heard of DF. Only 12% of the sample had good knowledge of DF. Those living in the lowlands were five times more likely to possess good knowledge than highlanders (*P*<0.001). Despite low knowledge levels, 83% of the people had good attitude and 37% reported good practice. We found a significantly positive correlation among knowledge, attitude and practice (*P*<0.001). Among the socio-demographic variables, the education level of the participants was an independent predictor of practice level (*P*<0.05), and education level and interaction between the sex and age group of the participants were independent predictors of attitude level (*P*<0.05).

**Conclusion:**

Despite the rapid expansion of DENV in Nepal, the knowledge of people about DF was very low. Therefore, massive awareness programmes are urgently required to protect the health of people from DF and to limit its further spread in this country.

## Introduction

Dengue virus (DENV) infection has globally become a major public health concern since the incidence of dengue fever (DF) has increased more than 30-fold over the last five decades [1] and the disease is now endemic in 128 countries [2]. According to a recent study [3], 390 million DENV infections are estimated to occur per year; over three times more than previous estimates by the World Health Organization (WHO) had suggested [1]. Despite progress with the development and clinical evaluation of vaccines against DENV infection, no such vaccine is on the market yet [4] and there is no specific treatment against DF. Thus, controlling the populations of DENV vector mosquitoes, especially *Aedes aegypti* and *Aedes albopictus*, and limiting their dispersal to new regions is crucial to prevent DENV transmission [5].

Over the last decade, the geographical distribution of DF has included new countries and more rural areas, making it the most rapidly expanding arboviral disease in the world [1]. In the WHO South-East Asia Region, where expansions of areas with DENV transmission have been documented in Bhutan and Nepal since 2004 and 2006, respectively [1], [5], the annual economic burden of DF was reported to be US$ 590 million (US$1.65 per capita) in 12 countries for the period of 2001–2010 [6]. Nepal was not included in that study, and information about the socio-economic aspects of DENV infection in this country is lacking.

In Nepal, DF is rapidly expanding its geographical range from south to north. The first case of DF was reported in a Japanese volunteer in 2004 [7], and the first isolation of DENV (serotype 2) was also made from a Japanese traveller to Nepal [8]. In 2006, the discovery, in southern lowland districts, of DF in patients without any history of travel to endemic areas along with the presence of the primary dengue vector mosquito *A. aegypti* confirmed the establishment of local DENV transmission in Nepal [9]. In addition, clinical and laboratory test results confirmed the circulation of all four DENV serotypes during the 2006 outbreak in Nepal [9]. This first DF outbreak in Nepal in 2006 led to the official recognition of local transmission in the country. The 32 confirmed cases of the 2006 outbreak were followed by 27 confirmed cases in 2007, 10 in 2008, 30 in 2009, and 917 including five deaths in 2010 when a major outbreak occurred in central Nepal [10], [11]. Although the travel history of cases reported from the capital of Nepal, Kathmandu, during the 2006 outbreak is not known, a case of DF without a history of travel to endemic areas was again reported from Kathmandu during the 2010 outbreak [12]. Kathmandu, a city with about one million susceptible inhabitants, is located in a mountain valley at around 1,300 m above sea level (asl). The two most important vectors of DENV infections, *A. aegypti* and *A. albopictus*, are established and widely distributed in the urban areas of this city [13], [14].

The impression of the major outbreak of DF in central Nepal in 2010, our findings regarding the distribution of DENV vectors in the capital of this Himalayan country [13], [14] and the lack of relevant studies on the knowledge, attitude and practice (KAP) of the people in Nepal regarding DENV transmission and DF prevention prompted us to conduct the present study. Our aim was to assess and compare the KAP of people residing in lowland and highland areas of Nepal, and to thereby guide and assist efforts to design information, education and communication (IEC) materials as well as behaviour change communication (BCC) programmes for dengue control in Nepal. In our present study, we have addressed the following major research questions:

Can differences in people's KAP of DF be explained by their socio-economic background?

Does the area of residence in Nepal (i.e., lowland and highland) affect the KAP level on DF?

## Methods

### Study design

A prospective study on *Aedes* mosquitoes and climate change along an altitudinal transect in central Nepal was designed to investigate the spatial and temporal distribution of dengue vectors and assess risk factors of DENV infection among inhabitants living in central Nepal. In this project, environmental scientists, entomologists, public health physicians, sociologists and biologists collaborated closely. As a part of this interdisciplinary project, we conducted a descriptive cross-sectional KAP study of households in three distinct ecological regions of Nepal: lowland (so-called terai), hill and mountain districts. Data were collected from September 2011 to February 2012. The study area cut through five administrative districts of central Nepal and covered a transect that extend along a major highway with an altitudinal range from 80 m to 2,100 m asl.

### Study site selection and its justification

The selected sites for this study were the Parsa, Makwanpur, Kathmandu, Nuwakot and Rasuwa districts of central Nepal. They represent broad vertical cross-sections of the three major ecological regions of Nepal. In the south, the study area starts in Birgunj (Parsa district; average altitude 88 m asl) at the southern border with India. It then extends to Hetauda (Makwanpur district), Kathmandu and Ranipauwa (Nuwakot district) in the hills, and to Dhunche (Rasuwa district; average altitude 2,050 m asl) in the mountain region bordering Tibet in the north. These sites were selected based on the existing road network from south to north, the mobility of the population and the level of urbanization which ranges from metropolitan (capital city) to rural areas in the mountain districts. The parts of the study area below 1,500 m asl were categorized as lowland (terai and hill region) and those above 1,500 m asl as highland areas (mountain areas). Furthermore, lowland and highland areas were based on known distribution of DENV vectors before the start of this study. The lowland areas included in this study are predominantly urban areas compared to the rural highlands areas, but all are densely populated and have good access to means of transportation and water supply. The key characteristics of each study site are given in Table 1.

**Table 1:** Key characteristics of study sites.

Characteristics	Birgunj	Hetauda	Kathmandu	Ranipauwa	Dhunche	*P*-value
Ecological region	Terai	Hill	Hill	Mountain	Mountain	NA
Urbanization	Urban	Urban	Urban	Rural	Rural	NA
Average altitude (m)	88	465	1310	1850	2050	<0.001
Category for our study	Lowland	Lowland	Lowland	Highland	Highland	
Total population^a^	139068	85653	1003285	7320	2744	<0.001
Total households^a^	24180	19890	254764	863	714	<0.001
Population density^a^ (per km^2^)	6569	1793	20289	NA	NA	<0.001
Number of motor vehicles*	1580	1033	23216	268	36	<0.001
Households with access to drinking water (%)* #	95.38	91.42	70.00	87.68	87.87	0.36
Households having radio (%)*	29.85	44.89	56.15	59.58	52.20	<0.05
Households having television (%)*	39.38	30.93	74.76	30.73	23.31	<0.001

a based on national population and housing census 2011.

*Taken as proxy from district indicator of census data 2011.

# Includes tap/piped water supply and tube-well/hand pump.

All *P*-values are based on chi-square analysis of numbers in each category.

NA means not available/applicable.

### Study sample and instruments

In the first phase, five districts from central Nepal were selected. In this selection, districts in which the major DF outbreak of 2010 had occurred were excluded because this experience could have biased the results of the study. In the second phase, we targeted 625 households (125 from each district) for the study employing a systematic sampling method. Five vector collection sites were randomly selected from each district for the KAP survey. Thereby, households located within a 50 m radius of each site were listed by doing a social mapping exercise and 25 households selected by systematic random sampling. One adult member was selected randomly from each household selected. Healthy individuals aged 15 years and above were included in the study. People having reported febrile illness and those who had migrated from other districts and countries within the past six months were excluded. Individual members of households (either the household head or a family member) were the study participants. The KAP questionnaire previously used in Jamaica [15] was adapted for our study. The questionnaire was modified for content, wording and cultural appropriateness following an extensive review of the literature published in English and expert opinions. The questionnaire consists of (1) socio-economic information (area of residence, age, sex, marital status, education level, religion and annual family income); (2) health information relating to whether the respondent might have had DF or not and sources of information; (3) knowledge of symptoms, signs and transmission modes of DENV infection; (4) attitude towards DF; and (5) preventive measures against DENV infection (e.g., methods used to reduce mosquito-human contact and mosquito breeding opportunities). As none of the study districts had a history of DF outbreak except a few reported cases from the lowlands, we excluded questions on the knowledge of DF management. Other changes to the content of the original questionnaire were minimal. The final draft of the questionnaire was translated into Nepali and re-translated into English to ensure that the meaning of the questions remained unchanged. Before its use in the main study, the questionnaire was pre-tested among the members of a community in Kathmandu which was not included in the final analysis. Cronbach's Alpha [16] was used to assess the reliability coefficient which is a measure of the internal consistency of the questionnaire. The result showed that Cronbach's Alpha coefficients of KAP domains were 0.82, 0.7 and 0.71 respectively. A minimum of 0.7 is considered to reflect acceptable reliability [17], [18]. Local university graduates were recruited and trained for data collection. The same individuals were involved throughout the data collection process in all districts. However, the interviewers were not aware of either the study hypotheses or correct answers to survey questions in order to avoid interviewer bias during data collection. Questions related to knowledge, attitude and practice were asked one-by-one sequentially to avoid bias. The interviewers also observed the availability of potential breeding places of mosquitoes in and around the house. These observations were further validated by a supervisor in the field.

### Ethical approval

The protocol of this study was approved by the Ethical Review Board (ERB) of the Nepal Health Research Council (NHRC). The objectives of the study were explained to local community people including community leaders and health professionals. Sufficient time was given to ask questions and it was emphasized that participation was voluntary and they could quit any time during the interview. As our research design involved no more than minimal risk and the literacy level of the majority of household heads was low leading to reluctance giving signatures or thumb prints on consent forms, oral informed consent in the presence of a local person or adult family member was obtained from all research participants before the beginning of any interview. In the case of minors, oral consent was first obtained from their household head (father or mother) and then from all child participants. Taking oral consent only was approved by the ERB in agreement with the National Ethical Guidelines for Health Research in Nepal [19].

### Survey data analysis

All completed questionnaires were double-checked and verified on the same day for completeness and consistency. The data was then entered into a database by trained personnel using the Epi Data 3.1 Software (EpiData Association, Denmark). All data files were checked and cleaned by two of the authors before analysis. The analysis of the data was performed in R computing software [20] using the “epicalc” package [21]. KAP assessment was executed using a scoring system. A participant's KAP score was computed as the sum of correct responses for the respective domain. The response was defined as correct if it was valid (i.e., supported by current literature; positive attitude; can identify appropriate measures to prevent mosquito breeding and DENV transmission). Each correct answer was scored 1 and wrong answers 0. We pooled “Do not know (DNN)” responses with wrong answers and scored them as 0 which is a conventional practice as “DNN” responses either come from the least knowledgeable respondents or the vast majority of those saying “DNN” really do not know [22]. Treating “DNN” as a wrong answer appears reasonable and justifiable in our study although it is a conservative strategy [23]. In contrast, dropping “DNN” responses from the data set reduces sample size, may introduce sample selection bias and result in serious loss of information [23]. Hence, we did not exclude “DNN” in the analyses. These scores were added to arrive at a single value out of a possible total score of 24 for knowledge, six for attitude and 20 for practice. Respondents' levels were defined as “good” or “poor” based on an 80% cut-off point. For example, with a total 24 of questions in the knowledge domain, a respondent securing 19 or more scores was categorized as having good knowledge level. In our survey, the non-response rate was 6%, and those data were not included in our analysis. Furthermore, individual non-response items were coded “NA” and were deleted during analysis in R. As only the income variable had missing responses (<5% of total), listwise deletion of “NA” items may not introduce bias in our analysis involving income.

The socio-economic composition of the samples included in the study was described as lowland, highland and overall. The KAP of people living in highland and lowland areas was compared using a Chi-square test. Fisher's exact test was used where more than 20% of the cells had expected cell counts less than five. Spearman's rank correlation (r_s_) was used to calculate correlation values between KAP scores because the KAP scores were not normally distributed as revealed by a Shapiro-Wilk normality test. We used Fisher's R- to- Z transformation to calculate confidence intervals (CI) and compare the correlations of KAP scores between highland and lowland areas. As Kathmandu is the densely populated metropolitan capital city of Nepal, we performed separate analyses excluding Kathmandu to compare highland and lowland areas. However, these additional analyses did not reveal significant differences.

Logistic regression analysis was carried out to identify determinants of the KAP level of DF. Independent variables included in the model were household location, household income, education level, and the age and sex of the respondent. A dependent variable introduced to the model was the separate KAP level (i.e., “good” vs. “poor”). Confounding factors were explored observing the difference between the adjusted odds ratio (aOR) in multivariate analyses and the crude odds ratio (OR) from univariate analyses of a particular independent variable with another independent variable. Determinants having a screening significance of *P<*0.25 in univariate analysis were selected for multivariate analyses [24]. The full model was fitted including all possible two-way interactions of selected variables from the univariate analyses. Model simplification was carried out using a stepwise procedure in R. In the first step, all two-way interactions were deleted and the reduced model was compared with the full model using a likelihood ratio test. If the deletion caused an insignificant change in deviance, the reduced model was selected; otherwise interaction terms were put back in the model using an update function. This process was continued removing the least significant terms from the model until the minimum adequate model was achieved. We also used Akaike's information criterion (AIC) to select the final model using an automated step function. When comparing two models, the smaller AIC was accepted as indicating the better fit [25]. Both procedures yielded exactly the same final model. We assessed multicollinearity of predictors for each model using variance inflation factors (VIFs). The VIFs for all predictor variables were less than 2.0. In the final model, we tested the main effects of determinants and the confounding interactions between them. All *P*-values are two tailed and were considered statistically significant at *P*<0.05.

## Results

### Socio-economic characteristics of the study population in central Nepal

Table 2 presents the socio-economic characteristics of the study sample as obtained during the KAP household survey. Out of a total of 625 households/individuals targeted for the study, 589 participants could be enrolled for the questionnaire survey with a response rate of 94%. Among the 589 participants interviewed using the structured questionnaire, 41% were residents of highland areas and 59% were from the lowlands. Of the total study participants, 41% were female and 59% were male. Most of the study participants were married (70%) and 29% were single. The median year of completed school education was grade 10; 12% of the participants were illiterate. The majority of the participants belonged to the Hindu religion (70%) followed by Buddhist (23%). Among the socio-demographic characteristics, only sex (*P*<0.05), annual income (*P*<0.01) and religion of participants (*P*<0.001) were significantly different between lowland and highland areas (Table 2).

**Table 2:** Socio-demographic characteristics of the study population in central Nepal in 2011/2012.

Socio-demographic characteristics	Highland n (%)	Lowland n (%)	Total n (%)	*P*-value
Age group				0.839
15–29	99 (41)	154 (45)	253 (43)	
30–44	86 (35)	114 (33)	200 (34)	
45–59	48 (20)	64 (18)	112 (19)	
60–84	10 (4)	14 (4)	24 (4)	
Sex				0.043
Female	111 (46)	128 (37)	239 (41)	
Male	132 (54)	218 (63)	350 (59)	
Marital status				0.65*
Divorced	0 (0)	2 (1)	2 (0)	
Married	175 (72)	236 (68)	411 (70)	
Single	65 (27)	103 (30)	168 (29)	
Widowed	3 (1)	5 (1)	8 (1)	
Education level				0.65
Illiterate	32 (13)	38 (11)	70 (12)	
Primary	27 (11)	44 (13)	71 (12)	
Secondary	99 (41)	153 (44)	252 (43)	
Higher education	85 (35)	111 (32)	196 (33)	
Religion				<0.001*
Buddhist	100 (41)	36 (10)	136 (23)	
Christian	2 (1)	2 (1)	4 (1)	
Hindu	135 (56)	279 (81)	414 (70)	
Muslim	6 (2)	29 (8)	35 (6)	
Annual income (NRS**)				0.006
<100,000	55 (31)	83 (32)	138 (32)	
100,000–250,000	118 (66)	145 (57)	263 (61)	
>250,000	5 (3)	27 (11)	32 (7)	

All *P*-values are based on chi-square analysis of numbers in highland and lowland categories except those indicated by an asterisk (*) which are based on Fisher's exact test.

**NRS means Nepalese Rupees.

### Knowledge of people about symptoms and signs of dengue fever

Only 455 (77%) of the research participants had previously heard about DF with significantly higher number in lowland compared to highland areas (*P*<0.001). The majority of participants (99% and 97%) were able to correctly identify general symptoms of DF like fever and headache, respectively, with no significant difference between highland and lowland areas. However, when further queried about the typical symptoms of DF, a significantly lower number of participants from the highland areas compared to the lowland residents were able to correctly identify these (*P*<0.05) (Table 3).

**Table 3:** Knowledge on symptoms and signs of dengue fever (DF).

Variables	Highland n (%)	Lowland n (%)	Total n (%)	*P*-value
Is fever a symptom of DF?				1*
No	1 (1)	3 (1)	4 (1)	
**Yes**	154 (99)	289 (99)	443 (99)	
Is headache a symptom of DF?				0.557*
No	3 (2)	9 (3)	12 (3)	
**Yes**	145 (98)	272 (97)	417 (97)	
Is joint pain a symptom of DF?				0.002
No	25 (25)	22 (11)	47 (16)	
**Yes**	74 (75	179 (89)	253 (84)	
Is muscle pain a symptom of DF?				0.003
No	25 (27)	23 (12)	48 (17)	
**Yes**	66 (73)	163 (88)	229 (83)	
Is pain behind the eyes a symptom of DF?				<0.001
No	38 (42)	28 (18)	66 (27)	
**Yes**	53 (58)	124 (82)	177 (73)	
Are nausea/vomiting symptoms of DF?				0.062
No	33 (31)	37 (21)	70 (24)	
**Yes**	73 (69)	143 (79)	216 (76)	
Is rash a symptom of DF?				0.018
No	33 (33)	31 (19)	64 (24)	
**Yes**	68 (67)	132 (81)	200 (76)	
Is diarrhea common in DF?				0.19
No	35 (37)	45 (28)	80 (31)	
**Yes**	60 (63)	115 (72)	175 (69)	
Is stomach pain common in DF?				0.047
No	31 (36)	38 (23)	69 (28)	
**Yes**	55 (64)	125 (77)	180 (72)	

All *P-*values are based on chi-square analysis of numbers in highland and lowland categories except those indicated by an asterisk (*) which are based on Fisher's exact test. Responses in bold font indicate correct answer.

### Knowledge of dengue virus transmission

Few participants (16%) knew that not all mosquitoes can transmit DENV and 19% knew that certain *Aedes* mosquitoes transmit DENV. Both responses were not significantly different between the highland and lowland study populations. On the other hand, more than half of the participants were aware of the fact that flies and ticks do not transmit DENV. Only 8% of the lowland and 5% of the highland participants knew that DENV transmitting *A. albopictus* and *A. aegypti* mosquitoes bite during day time, and only 8% of the lowland and 6% of the highland participants thought that they could identify these mosquitoes. Only responses about biting behavior were statistically significantly different by area of residence (*P*<0.001). About 92% of the participants responded that DF could be contracted through blood transfusion with significantly higher responses from highland compared to lowland areas (*P*<0.01) (Table 4).

**Table 4:** Knowledge of dengue fever (DF) transmission.

Variables	Highland n (%)	Lowland n (%)	Total n (%)	*P*-value
Can all mosquitoes transmit DF?				0.099
**No**	101 (79)	228 (86)	329 (84)	
Yes	27 (21)	37 (14)	64 (16)	
Do the *Aedes* mosquitoes transmit DF?				0.279
No	58 (85)	71 (77)	129 (81)	
**Yes**	10 (15)	21 (23)	31 (19)	
Do flies transmit DF?				0.475
**No**	85 (71)	161 (67)	246 (68)	
Yes	34 (29)	79 (33)	113 (32)	
Do ticks transmit DF?				0.043
**No**	52 (49)	142 (62)	194 (58)	
Yes	53 (51)	87 (38)	140 (42)	
Does ordinary person to person contact transmit DF?				0.004
**No**	48 (34)	122 (50)	170 (44)	
Yes	93 (66)	124 (50)	217 (56)	
Is DF transmitted through food and water?				0.104
**No**	59 (43)	131 (53)	190 (49)	
Yes	77 (57)	118 (47)	195 (51)	
Can DF be transmitted by blood transfusion?				0.002
No	3 (2)	31 (12)	34 (8)	
**Yes**	142 (98)	237 (88)	379 (92)	
When are the Dengue mosquitoes most likely to feed/bite?				<0.001
Both day and night	68 (43)	189 (63)	257 (56)	
**Day time**	7 (5)	24 (8)	31 (7)	
Night time	82 (52)	85 (29)	167 (37)	
Mosquitoes breed in standing water				0.14*
No	5 (3)	3 (1)	8 (2)	
**Yes**	150 (97)	277 (99)	427 (98)	
Windows screens and bed net reduce mosquitoes				0.505*
No	2 (1)	8 (3)	10 (2)	
Yes	154 (99)	288 (97)	442 (98)	
Insecticides sprays reduce mosquitoes and prevent DF				0.81
No	7 (5)	16 (6)	23 (5)	
**Yes**	146 (95)	269 (94)	415 (95)	
Tightly covering water containers reduces mosquitoes				0.027
No	25 (17)	26 (9)	51 (12)	
**Yes**	122 (83)	256 (91)	378 (88)	
Removal of standing water can prevent mosquito breeding				1*
No	4 (3)	9 (3)	13 (3)	
**Yes**	142 (97)	263 (97)	405 (97)	
Mosquito repellents prevent mosquito bites				0.401
No	8 (7)	24 (10)	32 (9)	
**Yes**	114 (93)	221 (90)	335 (91)	
Can you identify *Aedes* mosquitoes?				0.78
No	104 (94)	138 (92)	242 (93)	
**Yes**	7 (6)	12 (8)	19 (7)	

All *P-*values are based on chi-square analysis of numbers in highland and lowland categories except those indicated by an asterisk (*) which are based on Fisher's exact test. Responses in bold font indicate correct answer.

### Sources of information on dengue fever

**Figure 1:**
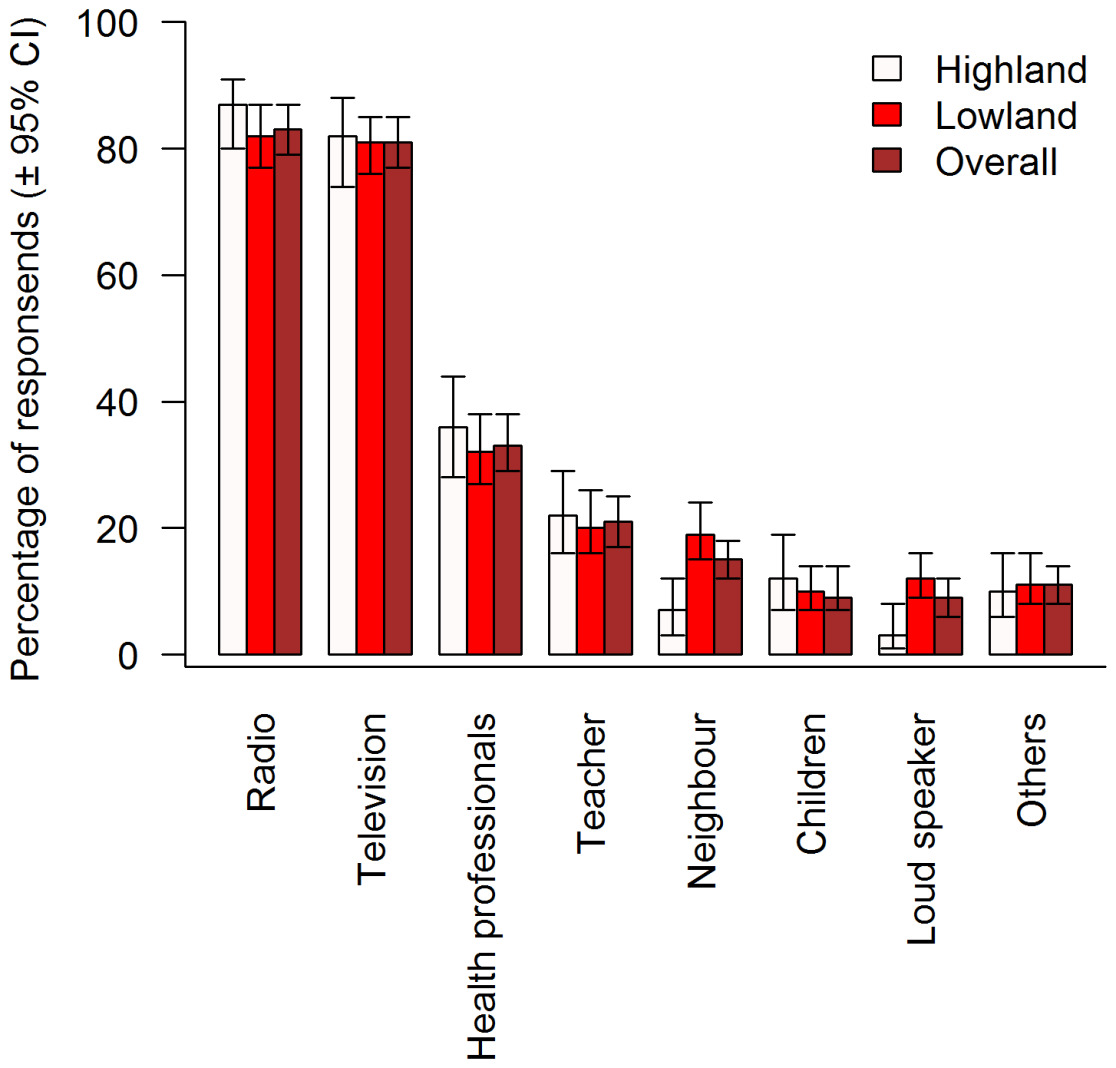
Sources of information on dengue fever. Error bars represent 95% confidence intervals.

Figure 1 presents findings on sources of information on DF. The majority of the research participants reported that they had heard of DF through the radio (83%) followed by television (81%). None of the sources of information were statistically significant between the highlands and lowlands except loud speaker announcement and neighbors (*P*<0.05) which were reported by more participants of lowland than highland areas. However, not much information was obtained from loud speaker announcement and neighbours (<20%).

### Attitudes towards dengue fever

Table 5 summarizes participants' attitudes regarding DF. Most of them strongly agreed (51%) or agreed (40%) that DF is a serious illness. Thus, 91% of the participants effectively appreciated the serious nature of the disease. None of the research participants strongly disagreed on facts related to DF. The attitude of people living in highland and lowland areas was significantly different (*P*<0.05) except for the statement that DF can be prevented.

**Table 5:** Attitudes towards dengue fever (DF).

Variables	Highland n (%)	Lowland n (%)	Total n (%)	*P*-value
Is DF a serious illness?				<0.001
**Strongly agree**	59 (38)	175 (59)	234 (51)	
**Agree**	86 (55)	98 (33)	184 (40)	
Not sure	7 (4)	14 (5)	21 (5)	
Disagree	5 (3)	11 (3)	16 (4)	
Are you at risk of getting DF?				0.013
**Strongly agree**	48 (31)	84 (28)	132 (29)	
**Agree**	45 (29)	127 (43)	172 (38)	
Not sure	24 (15)	40 (13)	64 (14)	
Disagree	40 (25)	47 (16)	87 (19)	
Can DF be prevented?				0.101*
**Strongly agree**	15 (10)	52 (17)	67 (15)	
**Agree**	131 (83)	231 (78)	362 (80)	
Not sure	9 (6)	12 (4)	21 (5)	
Disagree	2 (1)	3 (1)	5 (1)	
Is controlling the breeding places of mosquitoes a good strategy to prevent DF?				<0.001
**Strongly agree**	17 (11)	53 (18)	70 (15)	
**Agree**	131 (83)	210 (71)	341 (75)	
Not sure	3 (2)	31 (10)	34 (8)	
Disagree	6 (4)	4 (1)	10 (2)	
Do you think that stagnant water around the houses in discarded tyres, broken pots and bottles are breeding places of *Aedes* mosquitoes?				0.002
**Strongly agree**	19 (12)	61 (21)	80 (18)	
**Agree**	126 (80)	198 (66)	324 (71)	
Not sure	6 (4)	33 (11)	39 (9)	
Disagree	6 (4)	6 (2)	12 (2)	
Do you think communities should actively participate in controlling the vectors of DENV?				0.004*
**Strongly agree**	18 (11)	66 (22)	84 (19)	
**Agree**	131 (83)	206 (69)	337 (74)	
Not sure	5 (3)	22 (8)	27 (6)	
Disagree	3 (2)	4 (1)	7 (1)	

All *P*-values are based on chi-square analysis of numbers in highland and lowland categories except those indicated by an asterisk (*) which are based on Fisher's exact test. Responses in bold font indicate correct answer. DENV means dengue virus.

Note: None of the participants strongly disagreed with statements or questions in our study.

### Preventive measures against dengue fever

Table 6 shows that almost all respondents stated that preventing mosquito-man contact is the best strategy for the prevention of DF. The measures to reduce mosquito-man contact that were most commonly used by the majority of participants were: covering water containers in the home (95%), cutting down bushes in the yard (94%), eliminating standing water around the house (95%), disposing of water holding containers such as tyres, parts of automobiles, plastic bottles, cracked pots, etc. (91%), preventing any stagnant water (90%), cleaning out garbage/trash (92%), using window screens to keep mosquitoes out of the house (81%), using insecticide sprays (80%), turning containers upside down to avoid water collection (90%) and using mosquito coils (69%). Responses about preventive practices such as cutting down bushes in the yard, using mosquito coils, using fans and covering water containers in the home were significantly more common among lowland participants (*P*<0.05). Some responses on preventive measures that were less frequently given (e.g., using mosquito repellents/cream and using professional pest control to reduce mosquitoes) were significantly more common in highland areas (*P*<0.001).

**Table 6:** Preventive measures against dengue fever (DF).

Variables	Highland n (%)	Lowland n (%)	Total n (%)	*P*-value
Prevent mosquito-man contact				1*
No	1 (1)	2 (1)	3 (1)	
**Yes**	156 (99)	296 (99)	452 (99)	
Use insecticide sprays to reduce mosquitoes				0.759
No	29 (19)	60 (21)	89 (20)	
**Yes**	125 (81)	232 (79)	357 (80)	
Use professional pest control to reduce mosquitoes				<0.001
No	50 (40)	154 (65)	204 (56)	
**Yes**	74 (60)	84 (35)	158 (44)	
Use screen windows to reduce mosquitoes				0.503
No	33 (21)	54 (18)	87 (19)	
**Yes**	122 (79)	243 (82)	365 (81)	
Eliminate standing water around the house to reduce mosquitoes				0.175
No	12 (8)	12 (4)	24 (5)	
**Yes**	144 (92)	277 (96)	421 (95)	
Cut down bushes in the yard to reduce mosquitoes				0.043
No	15 (10)	13 (4)	28 (6)	
**Yes**	139 (90)	283 (96)	422 (94)	
Prevent water stagnation				0.077
No	21 (14)	23 (8)	44 (10)	
**Yes**	130 (86)	263 (92)	393 (90)	
Use mosquito eating fish to reduce mosquitoes				0.901
No	56 (71)	101 (73)	157 (72)	
**Yes**	23 (29)	38 (27)	61 (28)	
Use mosquito coils to reduce mosquitoes				<0.001
No	63 (43)	72 (25)	135 (31)	
**Yes**	83 (57)	218 (75)	301 (69)	
Cleaning of garbage/trash				0.07
No	17 (12)	18 (6)	35 (8)	
**Yes**	123 (88)	262 (94)	385 (92)	
Disposing water holding containers such as tires, parts of automobiles, plastic bottles, crack pots etc.				0.356
No	10 (7)	27 (10)	37 (9)	
**Yes**	144 (93)	255 (90)	399 (91)	
Use Mosquito repellent/cream				<0.001
No	68 (47)	179 (64)	247 (58)	
**Yes**	78 (53)	100 (36)	178 (42)	
Use of fan				<0.001
No	99 (69)	75 (26)	174 (40)	
**Yes**	44 (31)	217 (74)	261 (60)	
Use of smoke to drive away mosquitoes				0.313
No	88 (58)	183 (63)	271 (61)	
**Yes**	64 (42)	106 (37)	170 (39)	
Covering body with clothes				0.2
No	69 (45)	147 (51)	216 (49)	
**Yes**	86 (55)	139 (49)	225 (51)	
Do nothing to reduce mosquitoes				0.305
**No**	127 (85)	231 (81)	358 (82)	
Yes	22 (15)	55 (19)	77 (18)	
Cover water containers in the home				0.012
No	13 (9)	8 (3)	21 (5)	
**Yes**	139 (91)	284 (97)	423 (95)	
Frequently cleaning water filled containers and ditches around the house				0.662*
**Always**	73 (46)	139 (46)	212 (47)	
Never	3 (2)	2 (1)	5 (1)	
Often	44 (28)	89 (30)	133 (29)	
Sometimes	37 (24)	68 (23)	105 (23)	
Government sprays insecticides for controlling mosquitoes				0.253
No	69 (45)	147 (51)	216 (49)	
**Yes**	86 (55)	143 (49)	229 (51)	
Turning containers upside down to avoid water collection				0.768
No	15 (11)	24 (9)	39 (10)	
**Yes**	122 (89)	230 (91)	352 (90)	

All *P*-values are based on chi-square analysis of numbers in highland and lowland categories except those indicated by an asterisk (*) which are based on Fisher's exact test. Responses in bold font indicate correct answer.

### Correlation between knowledge, attitude and practice

The correlation of KAP scores overall revealed a significant positive correlation among knowledge-attitude (r_s_ = 0.39, *P*<0.001), knowledge-practice (r_s_ = 0.43, *P*<0.001) and attitude-practice (r_s_ = 0.26, *P*<0.001). However, the degree of correlation was fair (r_s_<0.5). The correlation between knowledge-practice only was higher in the highland compared to lowland (Table 7).

**Table 7:** Correlation between knowledge, attitude and practice scores.

Variables	r_s_ with 95%CI			
	Highland	Lowland	Total	*P*-value
Knowledge-attitude	0.37 (0.22–0.50)	0.40 (0.30–0.49)	0.39 (0.31–0.47)	0.726
Knowledge-practice	0.53 (0.41–0.63)	0.38 (0.28–0.47)	0.43 (0.35–0.50)	0.056
Attitude-practice	0.17 (0.01–0.32)	0.31 (0.20–0.41)	0.26 (0.17–0.34)	0.134

All *P*-values are based on Fisher's R- to- Z transformation of correlation coefficients in highland and lowland categories.

r_s_: Spearman rank correlation coefficients.

CI: Confidence intervals.

### Effect of socio-economic factors on the KAP level of dengue fever and its prevention

Regarding KAP scores, 12% of the participants achieved at least 80% on the knowledge score (good knowledge), 83% obtained at least 80% on the attitude score (good attitude) and 37% obtained at least 80% on the preventive practice score (good practice). In the univariate analysis of the associations between KAP and socio-economic variables of the study population (Table 8), we found increased odds of having good knowledge if the respondent lived in a lowland compared to highland area (OR: 5.07; 95% CI: 2.12–12.12). Similarly, we found increased odds of having a good attitude if respondents had completed higher education compared to illiterates (OR: 4.01; 95% CI: 1.59–10.12) and decreased odds of having a good attitude if they were older than 59 (60–84) compared to 15–29 years (OR: 0.15; 95% CI: 0.05–0.51). Furthermore, we found a significant association between preventive practices and the education level of the participants (*P*<0.05).

**Table 8:** Univariate logistic regression analysis showing predictors of knowledge, attitude and practice levels (good vs. poor).

Independent variable	Categories	Knowledge		Attitude		Practice	
		OR (95% CI)	*P*- value	OR (95% CI)	*P*- value	OR (95% CI)	*P*- value
Household location	Highland	1	<0.001	1	0.301	1	0.634
	Lowland	5.07 (2.12–12.12)		0.76 (0.45–1.29)		0.91 (0.61–1.35)	
Sex	Male	1	0.864		0.189		0.109
	Female	1.05 (0.59–1.87)		0.72 (0.44–1.17)		0.72 (0.49–1.08)	
Age group (years)							
	15–29	1	0.963	1	0.015	1	0.561
	30–44	0.97 (0.52–1.81)		0.71 (0.4–1.25)		0.87 (0.57–1.33)	
	45–59	1.1 (0.49–2.47)		0.53 (0.27–1.07)		0.74 (0.41–1.31)	
	60–84	1.64 (0.08–5.18)		0.15 (0.05–0.51)		0.5 (0.13–1.91)	
Education level							
	Illiterate	1	0.596	1	<0.001	1	0.031
	Primary	2.56 (0.5–13.21)		1.96 (0.66–5.77)		0.58 (0.4–11.35)	
	Secondary	2.01 (0.45–8.95)		1.3 (0.56–3.02)		1.32 (0.23–4.99)	
	Higher secondary	2.31 (0.52–10.29)		4.01 (1.59–10.12)		1.64 (0.23–5.03)	
Income level (NRS)							
	<100000	1	0.789	1	0.867	1	0.386
	100000–250000	0.88 (0.23–3.36)		1.14 (0.61–2.14)		1.32 (0.8–2.11)	
	>250000	1.22 (0.59–2.49)		1.32 (0.41–4.26)		0.85 (0.34–2.14)	

OR: Odds ratio.

CI: Confidence intervals.

NRS: Nepalese Rupees.

Table 9 shows determinants of practice and attitude of DF in multivariate logistic regression analysis. Although age and education level of the participants were significant predictors of attitude level in univariate analyses, after adjusting for confounding factors in multiple regressions, only education level and the interaction term of sex and age remained significant predictors in multivariate analyses. Study participants who had completed higher secondary education (aOR: 3.04; 95%CI: 1.11–8.35) were found to have a higher odds of good attitude compared to illiterates. Furthermore, females of older age group had significantly higher attitude scores than males. Similarly, education level only was a significant predictor of practice level. Participants who had completed higher secondary education (aOR: 1.68; 95%CI: 0.75–3.76) and secondary education (aOR: 1.32; 95%CI: 0.6–2.99) were found to have better practice compared to the illiterate group.

**Table 9:** Multiple logistic regression analysis showing predictors of attitude and practice levels (good vs. poor).

Dependent variable	Independent variable	Categories	OR (95% CI)	aOR (95%CI)	*P*-value
Attitude level	Sex	Male	1	1	1
			0.72 (0.44–1.17)	0.92 (0.41–2.03)	
	Age group	15–19	1	1	0.109
		30–44	0.71 (0.4–1.25)	0.91 (0.5–1.65)	
		45–59	0.53 (0.27–1.07)	0.74 (0.35–1.57)	
		60–84	0.15 (0.05–0.51)	0.2 (0.06–0.72)	
	Education level	Illiterate	1	1	0.006
		Primary	1.96 (0.66–5.77)	1.92 (0.63–5.83)	
		Secondary	1.3 (0.56–3.02)	1.07 (0.44–2.59)	
		Higher secondary	4.01 (1.59–10.12)	3.04 (1.11–8.35)	
	Sex: Age group	Male:15–19	1	1	0.048
		Female:30–44	0.29 (0.44–1.17)		
		Female:45–49	1.65 (0.32–8.42)		
		Female:60–84	3.41 (0.18–64.65)		
Practice level	Sex	Male	1	1	0.089
		Female	0.72 (0.49–1.08)	0.71 (0.47–1.06)	
	Education level	Illiterate	1	1	0.027
		Primary	0.58 (0.21–1.61)	0.58 (0.21–1.61)	
		Secondary	1.32 (0.59–2.93)	1.34 (0.6–2.99)	
		Higher secondary	1.64 (0.74–3.67)	1.68 (0.75–3.76)	

OR: Odds ratio.

aOR: Adjusted odds ratio.

CI: Confidence intervals.

## Discussion

After a decade with reported autochthonous DF cases followed by a series of outbreaks and a rapid expansion of the geographical range of DENV in Nepal [7], [9], [11], [12], [26], [27], there still was no study of the knowledge, attitude and practice aspects of DF and its control in this country except a single hospital based study from 2011 [28]. The findings of our present study suggest that there are relatively good attitudes and practices regarding DF control in Nepal despite a low level of knowledge. The knowledge level, on the other hand, was statistically significantly lower in highland than in lowland areas. Interestingly, the knowledge level was significantly lower in highland areas even when Kathmandu was analysed separately from the lowland areas. However, good practice levels were lower in the lowlands (albeit this difference was not statistically significant) indicating a higher risk of DF from this point of view. In all lowland study areas and the Kathmandu valley, the primary DENV vector *A. aegypti* had already been reported prior to this study [9], [10], [13]. In addition, *A. albopictus* is widely distributed in Nepal at least in the low and intermediate elevations including Kathmandu where it had been collected as early as the 1950s [13], [14], [29], [30]. Although its role in DENV transmission in Nepal remains to be elucidated, it is a known vector of DENV in other parts of the world [31]–[35].

The level of knowledge on DF reported in this study is comparable to that found in similar KAP studies conducted in India, Pakistan, Thailand and Jamaica [15], [36]–[40]. Most respondents were not able to correctly identify typical symptoms of DF apart from a few who identified fever and headache as obvious symptoms. Fever and headache were also the most frequently stated symptoms in similar studies conducted in India, Thailand, Laos, Nepal, the Philippines and Jamaica [15], [28], [36], [37], [39]–[41]. The most common clinical signs and symptoms reported during the 2010 DF outbreak in central Nepal included fever (100%), headache (97%), body ache (93%), nausea (85%), vomiting (63%), retro-orbital pain (49%), itching (43%), abdominal pain (42%), skin rashes (27%) and diarrhoea (26%) [11]. Presumably the participants in our study could not state typical symptoms of DF because they had not personally experienced the disease nor witnessed a case from a close relative or member of the community. The poor knowledge of symptoms associated with DF among our study population means that this disease may easily be confused with other common causes of fever such as influenza, typhoid, etc., inviting delays for check-up until complications develop as reported in a study from Jamaica [15]. A possible reason for this low level of knowledge on DF may be that there have been no regular awareness programmes on this infection except at the time of the 2010 outbreak [42] and that the disease itself only recently emerged in the country.

Knowledge on the transmission pathways of DENV was low. The dengue vectors *A. aegypti* and *A. albopictus* bite mostly after dawn and before dusk [43], [44]. Although *A. aegypti* usually does not bite at night, it will feed at night in lighted rooms [45]. However, only about 7% of the research participants were aware of this unique day biting habit of the DENV vectors. *Aedes aegypti* is a nervous feeder which may consecutively feed on more than one person, increasing its epidemic efficiency by infecting many members of a family [46]. Although DENV is transmitted by the bites of infected *A. aegpyti* and *A. albopictus*, only 19% of the respondents stated that *Aedes* mosquitoes transmit DENV and only 7% could identify such mosquitoes. In contrast to ours, a study from Laos showed that about 93% of the participants knew that *A. aegypti* was the specific mosquito species that transmits DENV [40]. More importantly, more than 56% of the participants in our study were not aware of the fact that ordinary person to person contact does not transmit DENV, highlighting the need for proper education about the protection from *Aedes* mosquito bites and the care of community members suffering from DF.

Interestingly, 92% of the participants stated that DF could be contracted through blood transfusion. Although, transmission of DENV by blood transfusion was not documented in the past, there is increasing evidence of DENV transmission by blood products [47]–[50] suggesting an additional risk to blood safety in DF endemic countries. For example, an estimated risk of DENV ranging from 1.6 to 6 per10,000 blood transfusions in Singapore during 2005 [50] and an average estimated risk of viremic donations of 7 per 10,000 in Puerto Rico over a period of 1995 to 2010 [47] were reported.

Our study shows that only 77% of the respondents had heard of DF, and that this proportion was significantly higher in the lowlands (*P*<0.001). In a multi-country study conducted in India, Indonesia, Myanmar, the Philippines and Thailand, the vast majority of respondents (>90%) had heard of the disease except for a few in the Philippines [51]. Most respondents in our study (>80%) reported that radio and television had been their predominant sources of information on DF followed by health professionals. Similar findings were reported from Jamaica [15], Laos [40] and the Philippines [41]. In Pakistan, the major source of information on DF was reported to be television followed by newspapers and relatives/friends/family [52]. In the present study only about one third of the participants had received information about DF from health professionals. This indicates that health professionals in Nepal are not adequately mobilized for awareness raising programmes and thus IEC materials need to be developed and distributed so that health workers can maximize the benefits of health facility visits by also communicating correct information about DF and its prevention.

Regarding attitude towards DF control, the majority of participants (82%) in our study were classified as having good attitude with a statistical non-significant higher proportion in the highlands (85% vs. 82%). This shows that the majority of people had perceived a risk of DF and seemed supportive towards DF control. However, this result might also be partially influenced by the Nepalese culture of trying to please the enumerators, who are regarded as guests, by agreeing or strongly agreeing to interview questions. In fact, none of the respondents indicated that they strongly disagreed with any of the statements, possibly due to the same cultural context. Similar findings on the attitude of people on DF risk and possible cultural influence was reported in a study in Malaysia [53]. Nevertheless, frequent DF outbreaks in the lowlands and an increasing mosquito nuisance in the highlands in recent years might have triggered a higher risk perception of DF in this study.

In our study, a translation of knowledge and attitude into practice was clearly observed. For example, more than 90% of the participants each from lowland and highland areas stated that stagnant water around the houses is the breeding source of mosquitoes and that controlling the breeding places of mosquitoes is a good strategy to prevent DF. They consequently also stated in the practice section that eliminating standing water around the house and disposing of water holding containers such as tyres, parts of automobiles, plastic bottles, cracked pots, etc., was useful to reduce the number of mosquitoes. It was not possible in our study to determine how all these reported practices were translated into actual practice. In similar studies conducted in Malaysia a discrepancy between knowledge and practice was reported [38], [53]. However, the observations made by the interviewers and the supervisory team of the present study suggested that there were very few suitable breeding places around the houses and that the yards were kept clean better in highland compared to lowland areas, supporting the validity of the reported practices.

Overall, in our study, the good practice level was many times higher than the good knowledge level. This sharply contrasts with similarly unexpected findings in a study in Thailand where persons with knowledge of DENV vector breeding sites had more potential breeding sites in and around their houses than persons without such knowledge [24]. In another study from northern Thailand, the reported use of preventive measures was also found to be higher than the reported knowledge of preventive measures [36]. One of the reasons for the higher practice levels attained in this study may be that many questions on the practice level were related to daily practices for the control of other mosquito-borne diseases and mosquito nuisance in general whereas questions on the knowledge dimension were very DF-specific, and many of the research participants had neither had DF themselves nor seen an *A. aegypti* mosquito. Our findings on practice levels are contrary to those of other studies which reported high levels of knowledge but low levels of practice [15], [24], [53] but consistent with those from a hospital-based study conducted in the lowlands of Nepal [28] and a study in northern Thailand [36]. Nevertheless, only about one third of the participants of our study reported good practices for DF prevention. We could not observe any significant difference between the KAP levels of highland and lowland areas even when Kathmandu was excluded from the lowland areas in the analyses.

In our KAP study we found that good knowledge on DF and its vector mosquitoes was scarce, good practice levels fairly high, and a good attitude towards DF control very common. Among all socio-demographic variables, the overall knowledge of the participants was significantly associated only with the area of residence (i.e., highland or lowland). There was no significant association of knowledge with sex, age or education level of the participants, a finding consistent with results from Malaysia [53]. This may be explained by the fact that formal school and university curricula in health education do not have contents on DF in Nepal and respondents obtained information elsewhere (e.g., from radio and television). A significant association was observed between attitude and the socio-demographic factors age and education level of the respondents. Attitude was negatively associated with increasing age, being significantly worse in the 60–84year age group. It increased with education level and was significantly better in respondents who had completed higher secondary education.

We found a significant association between the practice of controlling DENV transmission and socio-economic factors. Education level was associated with good practices among the respondents who had completed secondary or higher secondary education. In comparing this result with those of other countries, our findings are consistent with those from a study in Thailand [24]. On the contrary, a lack of significant association between socio-economic factors and practice level was observed in Malaysia and Jamaica [15], [53], possibly because the practice in the community is influenced mainly by local tradition and culture. However, practice levels were better in highland compared to lowland areas of Nepal despite the high risk of DF in the lowlands. As many breeding places such as discarded tyres and stagnant water are common in the lowlands where mosquitoes have been a nuisance for ages, lowland people might have ignored preventive practices. In contrast, mosquito nuisance only recently appeared in many highland areas of Nepal [54], and people there show a greater interest in controlling mosquito breeding places to avoid bites. This can also be observed in a higher correlation of knowledge and practice in the highlands (Table 7). Thus, poor practices along with a high density of the human population, movement of people and goods, and a suitable environment for the perennial distribution of DENV vectors in lowland Nepal might contribute to the greater risk of transmission there.

Only the area of residence of participants was found to be an independent predictor of knowledge levels in the study population. Participants living in lowland areas were five times more likely to possess good knowledge compared to those living in highland areas. The higher knowledge level among study participants in the lowlands may be due to relatively frequent DF outbreaks in the lowlands of Nepal in recent years [9], [26], [27], [55] and occasional awareness programmes at the time of outbreaks [42]. The higher attitude level among older female participants may be attributed to the major role of women in domestic works, collecting and storing water for domestic use, caring of ill members of the family and cleaning the breeding places of mosquitoes and to the fact that DENV vectors are primarily domestic and peri-domestic breeders [56], [57]. Furthermore, people who had completed primary or further education had a higher probability of good attitude, and those who had completed secondary and further education had significantly higher good practice levels. This underscores how important education is for changing the attitude and practice levels of people. Sex of participants was a significant predictor of knowledge in a study conducted in northern Thailand [36], and education of practice level in another study from Thailand [24]. In contrast to our findings, a study conducted in Pakistan identified only income and type of hospital presented to as independent predictors of knowledge [52]. We predict that education will make people more aware of their health and related health seeking behaviours such as the control of risk factors of diseases and cooperation in order to tackle community health problems. We found a significant positive correlation between the knowledge, attitude and practice of respondents in our study. Furthermore, the correlation between knowledge and practice only was higher in highland compared to lowland areas. No significant correlation between knowledge about DF and preventive practice was reported in a study from Jamaica [15].

We did not include any lowland districts where DF outbreaks had occurred in 2010 in order to avoid biases such as higher knowledge, attitude and practices among the participants whose family members or neighbours had had DF or been subjected to the awareness campaigns that had been organized in outbreak areas. However, omitting outbreak districts might also constitute a missed opportunity, as it would have allowed for interesting comparisons with non-outbreak districts. Nevertheless, a hospital-based study conducted after the DF outbreak in 2010 suggested better knowledge and attitude among people from outbreak districts, but this difference was not significant [28]. We also conducted separate analyses excluding Kathmandu from the lowland area data under the assumption that Kathmandu was the capital and metropolitan city of Nepal and hence might affect the comparison. However, the exclusion of Kathmandu did not change our findings.

The results of our study must be interpreted with caution because the study was cross-sectional, assessed relationships based on one point in time and did not account for the dynamics of relationships between the factors analysed. More importantly, it is possible that some respondents might have provided socially desirable responses to some questions [58], especially in the attitude domain, since the survey was conducted by an interviewer-based use of a structured questionnaire. Thirdly, we collected data along the highway in densely populated urban and semi-urban areas of one altitudinal transect within a 50 m radius from the vector collection sites only which may not be representative for the whole country. However, this study provides crucial baseline information on the overall KAP of people regarding DF and on relevant differences between people living in highland and lowland areas of Nepal.

## Conclusions

We found a low level of good or sufficient knowledge on DF in our sample population based on overall scores. Despite this low level of knowledge, the practice level was fair and attitude level very good. Compared to people in the highlands, lowland people are at particularly high risk because they were found to have lower practice levels and DENV vectors are widely established in the lowlands of Nepal. Therefore, there is an urgent need for massive awareness programmes to raise the knowledge of community people in Nepal. For this, social mobilization and communication programmes could be developed. These can be achieved through the development of IEC/BCC programmes on DF and the use of radio and television for broadcasting messages on DENV vector control and orienting more health professionals, school teachers and community leaders about these topics. The mobilization of female community health volunteers will be very important in this context as they have very good networks at the household level and represent the largest work force of the health sector in Nepal. Most importantly, the inclusion of DF and its prevention and control should be promoted in school and university curricula to raise awareness among students and use them as multipliers. This is important because the good knowledge level among literate people was also low in our study although good attitude and practice levels were higher compared to illiterate people or those who had only completed primary education. Such programmes will be crucial to win community support towards adopting effective measures for preventing DENV transmission, improving surveillance and healthcare-seeking behaviour, and better controlling outbreaks.
