# Correction: Knowledge, Attitude and Practice Regarding Dengue Fever among the Healthy Population of Highland and Lowland Communities in Central Nepal

**DOI:** 10.1371/journal.pone.0110605

**Published:** 2014-10-06

**Authors:** The PLOS ONE Staff

There is an error in affiliation 3 for author Meghnath Dhimal. Affiliation 3 should be: Institute for Atmospheric and Environmental Sciences (IAU), Goethe University, Frankfurt am Main, Germany.
